# A simple scoring system for the prediction of early pregnancy loss developed by following 13,977 infertile patients after in vitro fertilization

**DOI:** 10.1186/s40001-023-01218-z

**Published:** 2023-07-14

**Authors:** Yan Ouyang, Yangqin Peng, Senmao Zhang, Fei Gong, Xihong Li

**Affiliations:** 1 Reproductive and Genetic Hospital of CITIC-Xiangya, Changsha, China; 2 Clinical Research Center For Reproduction and Genetics in Hunan Province, Changsha, China; 3 Department of Epidemiology and Health Statistics, Xiangya School of Public Health, Central South University, Changsha, China

**Keywords:** Miscarriage, Early pregnancy loss, In vitro fertilization-embryo transfer, Transvaginal sonography, Scoring system, Prediction

## Abstract

A retrospective study was conducted to investigate a convenient simple scoring system for the prediction of early pregnancy loss (EPL) based on simple demographics. A total of 13,977 women undergoing transvaginal ultrasound scans on Days 27–29 after in vitro fertilization-embryo transfer (IVF-ET) from June 2016 and December 2017 were included. The first trimester pregnancy outcome was recorded at 12 weeks of gestation. The areas under the curve of this scoring system were 0.884 (95% confidence interval (CI) 0.870–0.899) and 0.890 (95% CI 0.878–0.903) in the training set and test set, respectively. The score totals ranged from -8 to 14 points. A score of 5 points, which offered the highest predictive accuracy (94.01%) and corresponded to a 30% miscarriage risk, was chosen as the cutoff value, with a sensitivity of 62.84%, specificity of 98.79%, positive predictive value (PPV) of 88.87% and negative predictive value (NPV) of 94.54% for the prediction of EPL in the training set. In the test set, a score of 5 points had a sensitivity of 64.69%, specificity of 98.78%, PPV of 89.87% and NPV of 93.62%, and 93.91% of the cases were correctly predicted. Therefore, the simple scoring system using conventionally collected data can be conveniently used to predict EPL after ET. However, considering the limitations, its predictive value needs to be further verified in future clinical practice.

**Supplementary Information:**

The online version contains supplementary material available at 10.1186/s40001-023-01218-z.

## Introduction

The first routine ultrasound scan is commonly performed on Day 28 after in vitro fertilization-embryo transfer (IVF-ET) in most reproductive centers in China to determine the location and viability of the embryo. Although the appearance of cardiac activity at first scan means a higher likelihood of continuing pregnancy, the rate of subsequent miscarriage is between 2 and 16% [1], and there are some examples of pregnancies that were initially thought to have poor viability but eventually developed normally [2]. The mental and psychological pressure on women undergoing IVF-ET is significant, especially when the viability of a pregnancy is uncertain. Under these circumstances, the physician has an important role in rapidly and accurately predicting the pregnancy outcome in an evidence-based and professional manner, as this would be helpful for further consultations and in determining subsequent management.

Individual maternal factors, such as a high maternal age (MA) [3], and abnormal ultrasound parameters, such as embryonic bradycardia and excessively large or small yolk sac diameter (YSD), have been shown to be associated with pregnancy failures in previous studies [4, 5]. Some previous mathematical models involve combinations of individual risk factors and exhibit reasonable performance [2, 6–9]. However, these models also have some shortcomings, such as an insufficient sample size in most studies, study groups being mixed (natural and assisted pregnancies), inconsistent gestational age (GA), and studies not providing specific probabilities of ongoing pregnancy or miscarriage.

In addition, many patients will return to the local hospital after pregnancy. We aimed to build a model by using ultrasound data obtained from real-time measurements and simple clinical indicators such as MA to quickly predict and assess pregnancy in different hospitals without knowing many of the patient’s assisted fertility indicators.

Therefore, the aim of this study was to investigate a convenient and accurate simple scoring system for the prediction of early pregnancy loss (EPL) based on simple demographics and the first routine ultrasound scan performed on days 27–29 after ET.

## Methods

### Participants

This retrospective study was conducted at the Reproductive and Genetic Hospital of CITIC-Xiangya (Changsha, Hunan, People's Republic of China). The institutional review board approved this study (date of approval: 26 July 2019; reference number: LL-SC-2019-015; Changsha, China).

The infertile patients included in this study underwent IVF treatment between June 2016 and December 2017. One to 2 embryos with good quality were transferred at Day 3 or Day 5. The embryo morphology was scored according to the criteria by Hardarson et al. [10]. The first routine transvaginal sonography (TVS) scan using a 5–9 MHZ probe (GE VOLUSON 730 or E8, General Electric) was arranged on days 27–29 to observe the number of embryos and their locations and viability. Only intrauterine singleton pregnancies were included. Measurements were taken in accordance with the ISUOG practice guidelines [11] and conformed to uniform standards: gestational sac diameter (GSD) was calculated as the mean value of 3 perpendicular diameters with the calipers placed at the inner edges of the trophoblast; YSD was calculated as the average of 3 perpendicular diameters with the calipers placed at the center of the yolk sac (YS) wall; embryonic length (EL) was measured as the greatest length of the embryo in the anterior to posterior dimension; and embryonic heart rate (EHR) was calculated from frozen M-mode images with electronic calipers by measuring the distance between two heart waves. The presence of intrauterine hematoma (IUH), a hypoechoic or anechoic crescent-shaped area between the chorionic membrane and the myometrium, was also noted. Data on clinical characteristics including day-14 (blastocysts on day 12), serum β-human chorionic gonadotropin (HCG) levels, MA, duration of infertility, infertility type and endometrial (EM) thickness on transfer day were also collected. The first-trimester pregnancy outcomes of these participants were noted at 12 weeks of gestation. Women with a continuing pregnancy for > 12 weeks of gestation were classified as ongoing pregnancy, and women with spontaneous miscarriage before or at 12 weeks of gestation were classified as EPL.

### Statistical analysis

Categorical variables are described using frequencies, and continuous variables are described using means and standard deviations. Pearson’s chi-squared test, Fisher’s exact test, T-test or the Wilcoxon rank sum test was used to compare categorical or continuous variables between women with ongoing pregnancies and women with EPL. The cases from the first year (year 2016) were used to generate the training set, and the cases from the second year (year 2017) were used as the verification set. The binary logistic regression (LR) model was used in the training set to identify the probable predictive factors of EPL. Points associated with each category of each risk factor were computed, and the risks associated with point totals were determined according to the “The Framingham Study risk score system” [12]. Based on the area under the curve (AUC) and the clinical value, we evaluated the cutoff value of the prediction model that had a relatively high risk of miscarriage and high prediction accuracy. Then, we used the verification set to evaluate and verify the simple scoring system. All statistical analyses were performed using SPSS version 24.0 software (SPSS, Inc.). The results of each test were considered significant when the two-sided P value did not exceed 0.05, except where otherwise specified.

## Results

From June 2016 to December 2017, 23,929 infertile patients conceived clinical pregnancies via IVF-ET in our hospital. A total of 14,118 women with an intrauterine singleton pregnancy were identified during this period and among them, 141 patients were lost to follow-up. Finally, a cohort of 13,977 women were enrolled, including 12,051 patients with ongoing pregnancies and 1926 patients with EPLs.

### Comparisons of women with ongoing pregnancies and EPLs

Compared with women with ongoing pregnancies, women with EPLs had significantly higher MA, body mass index, infertility duration and transfer cycle and significantly lower Day 14 hCG and EM thickness on transfer day (p < 0.001). The infertility type, cause of infertility, and insemination methods were also significantly different between women with ongoing pregnancies and women with EPLs (p < 0.05). The number of embryos transferred was not significantly different (p = 0.44).

Based on the TVS measurements, the GSD (18.5 ± 3.6 vs. 13.2 ± 4.8 mm), EL (3.5 ± 0.9 vs. 1.2 ± 1.6 mm), YSD (3.6 ± 0.4 vs. 2.6 ± 1.5 mm) and EHR (114.5 ± 2.2 vs. 42.4 ± 53.5 bpm) were significantly greater in women with ongoing pregnancies than those with EPLs (p < 0.001). The incidence of IUH (16.0% vs. 18.8%, P = 0.002) was also markedly higher in women with EPLs (Table 1).

**Table 1:** Comparisons of the parameters between 2 groups

Parameter	Ongoing pregnancy (12,051)	Early pregnancy loss (1,926)	P
MA (years)	30.9 ± 4.5	33.3 ± 5.4	< 0.001
BMI(kg/m2)	21.8 ± 2.4	22.1 ± 2.6	< 0.001
Infertility duration (years)	4.0 ± 3.0	4.4 ± 3.6	0.001
Transfer cycle	1.2 ± 0.7	1.4 ± 0.8	< 0.001
Infertility type			
Primary	4673 (38.78%)	602 (31.26%)	< 0.001
Secondary	7378 (61.22%)	1324 (68.74%)	
Cause of infertility			
Male	831 (6.90%)	112 (5.82%)	0.014
Female	6920 (57.42%)	1176 (61.06%)	
Combined male and female	3764 (31.23%)	550 (28.56%)	
Unexlained	536 (4.45%)	88 (4.57%)	
14-day HCG (mIU/ml)	594.4 ± 301.7	430.5 ± 283.9	< 0.001
EM thickness on transfer	12.6 ± 2.0	12.3 ± 1.9	< 0.001
Insemination methods			
IVF	5584 (46.34%)	829 (43.06%)	< 0.001
ICSI	2081 (17.27%)	293 (15.22%)	
IVF/ICSI	4386 (36.40%)	804 (41.72%)	
Number of embryos transferred	1.7 ± 0.5	1.7 ± 0.5	0.44NS
Intrauterine hematomas			
Presence	1928 (16.00%)	363 (18.85%)	0.002
Absence	10,123 (84.00%)	1563 (81.15%)	
GSD (mm)	18.5 ± 3.6	13.2 ± 4.8	< 0.001
YSD (mm)	3.6 ± 0.4	2.6 ± 1.5	< 0.001
EL (mm)	3.5 ± 0.9	1.2 ± 1.6	< 0.001
EHR (bpm)	114.5 ± 12.2	42.4 ± 53.5	< 0.001

*MA* maternal age; *BMI* body mass index; *HCG* human chorionic gonadotropin; *EM* endometrium; *IVF* in vitro fertilization; *ICSI* intracytoplasmic sperm injection; *NS* not significant; *GSD* gestational sac diameter; *YSD* yolk sac diameter; *EL* embryonic length; *EHR* embryonic heart rate

### Binary LR analysis

**Fig. 1:**
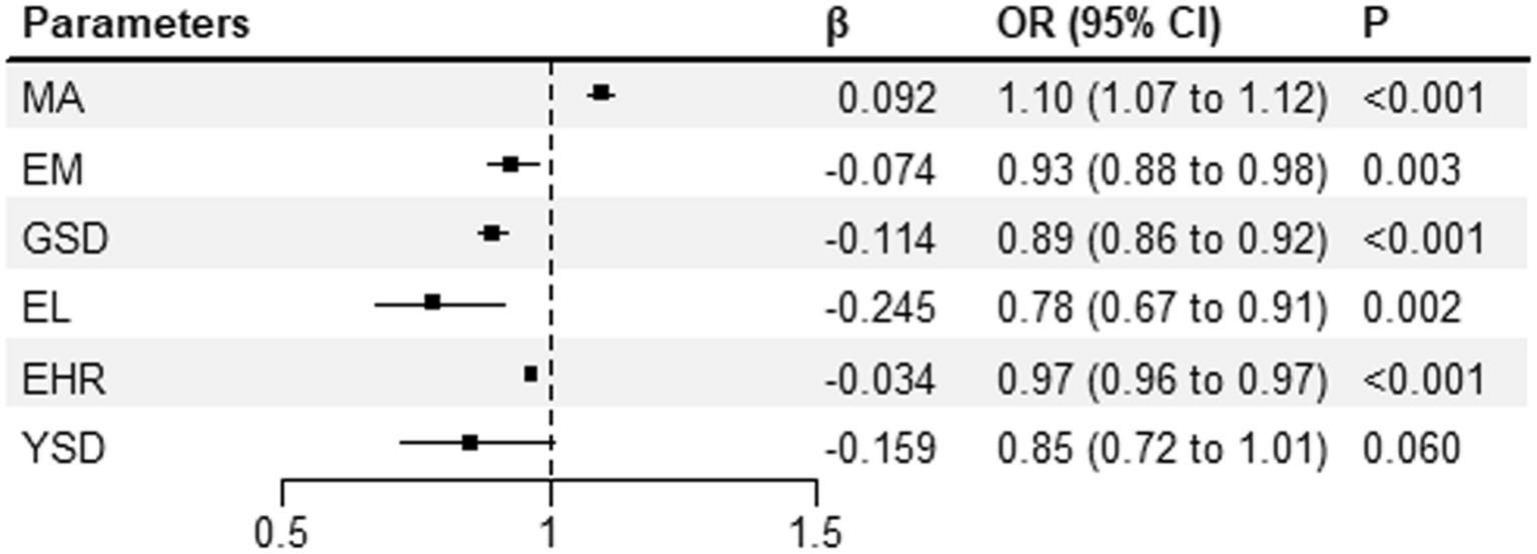
Predictive factors for EPL. *EPL* early pregnancy loss; *OR* odds ratio; *CI* confidence interval; *MA* maternal age (years); *EM* endometrium (mm); *GSD* gestational sac diameter (mm); *EL* embryonic length (mm); *EHR* embryonic heart rate (bpm); *YSD* yolk sac diameter (mm)

After stepwise screening, MA (p = 0.0001, OR 1.096, 95% confidence interval (CI) 1.073–1.119), GSD (p = 0.0001, OR 0.892, 95% CI 0.864–0.921), EL (p = 0.0020, OR 0.783, 95% CI 0.672–0.913), YSD (p = 0.0600, OR 0.853, 95% CI 0.723–1.007), EHR (p = 0.0001, OR 0.966, 95% CI 0.961–0.971) and EM on transfer day (p = 0.0030, OR 0.929, 95% CI 0.884–0.976) were found to be predictive factors of EPL and finally entered the scoring system (Fig. 1).

### The scoring system

The points associated with each of the categories of the risk factors were calculated and are shown in Table 2. Table 3 shows the scoring system derived from the binary LR model and the predictive value of each total point. The AUC of this scoring system was 0.884 (95% CI 0.870–0.899) in the training set. The score totals ranged from -8 to 14 points. Scores of 5 and 6 points offered the highest predictive accuracy (94.01% and 93.95%, respectively), and the predicted risks of miscarriage were 30.03% and 39.55%, respectively. Considering that women undergoing IVF were anxious, we chose a score of 5 points corresponding to 30.03% risk of miscarriage as the cutoff value, with a sensitivity of 62.84%, specificity of 98.79%, positive predictive value (PPV) of 88.87% and negative predictive value (NPV) of 94.54%, for the prediction of EPL in the training set.

**Table 2:** Points associated with each of the risk factor categories

Variable	Reference value (W_ij_)	*β*_*i*_	*β*_*i*_ (W_ij_-W_iREF_)	Points
MA (years)		0.0920		
< 29	26.00		− 0.78	− 2
30–39	34.50 = W_1REF_		0.00	0
≥ 40	42.00		0.69	2
GSD (mm)		− 0.1150		
< 9.0	6.20		1.47	3
9.0–12.9	11.00		0.91	2
13.0–27.0	19.00 = W_2REF_		0.00	0
> 27.0	27.30		− 0.95	− 2
EL (mm)		− 0.2450		
< 2.0	1.00		0.78	2
2.1–6.3	4.20 = W_3REF_		0.00	0
> 6.3	6.50		− 0.56	− 1
EHR (bpm)		− 0.0340		
< 100	50.00		2.21	5
100–130	115.00 = W_4REF_		0.00	0
≥ 130	140.00		− 0.85	− 2
YSD (mm)		− 0.1590		
< 3.00	1.50		0.40	1
3.00 ~ 4.99	4.00 = W_5REF_		0.00	0
≥ 5.00	6.00		0.32	1
EM (mm)		− 0.074		
< 10.0	5.00		0.56	1
10–14.99	12.50 = W_6REF_		0.00	0
≥ 15.00	16.30		− 0.28	− 1

Points = β_i_ (W_ij_−W_iREF_)/B B = 5*0.0920

*MA*  maternal age; *GSD* gestational sac diameter; *EL* embryonic length; *EHR* embryonic heart rate; *YSD* yolk sac diameter; *EM* endometrium

**Table 3:** Scoring system derived from the binary LR model

Sum of points	Estimated risk of miscarriage (%)	Sensitivity (%)	Specificity (%)	PPV (%)	NPV (%)	ACC (%)
− 8	0.18	100.00	0.00	13.30	−	13.30
− 7	0.24	100.00	0.00	13.30	−	13.30
− 6	0.31	100.00	0.00	13.30	−	13.30
− 5	0.54	100.00	0.00	13.30	−	13.30
− 4	0.81	100.00	0.11	13.32	100.00	13.40
− 3	1.24	99.69	0.79	13.36	94.34	13.95
− 2	1.85	99.28	5.32	13.86	97.95	17.82
− 1	2.84	93.89	34.88	18.12	97.38	42.74
0	4.20	91.72	46.37	20.79	97.33	52.40
1	7.33	80.23	83.80	43.18	96.51	83.32
2	11.02	75.98	91.74	58.53	96.14	89.64
3	15.57	70.91	96.22	74.21	95.57	92.85
4	22.03	68.01	97.55	81.01	95.21	93.62
5	30.03	62.84	98.79	88.87	94.54	94.01
6	39.55	66.87	98.11	84.44	95.07	93.95
7	50.59	57.04	99.02	89.89	93.76	93.43
8	60.50	49.48	99.41	92.82	92.77	92.77
9	70.67	39.13	99.57	93.33	91.42	91.53
10	78.54	26.71	99.83	95.91	89.87	90.10
11	84.90	15.63	99.92	96.79	88.53	88.71
12	89.51	6.11	99.95	95.16	87.40	87.47
13	92.65	3.00	100.00	100.00	87.04	87.10
14	95.42	0.52	100.00	100.00	86.76	86.76

*LR* logistic regression; *PPV* positive predictive value; *NPV* negative predictive value; *ACC* accuracy

In the verification set, the AUC of the scoring system was 0.890 (95% CI 0.878–0.903). A score of 5 points had the highest diagnostic accuracy, with 93.91% of the samples correctly predicted. The sensitivity, specificity, PPV and NPV were 64.69%, 98.78%, 89.87% and 93.62%, respectively (Table 4, Additional file 1: Table S1).

**Table 4:** The classification of the results obtained using a score of 5 as the cutoff value

Training samples	Scoring system		Total
	EPL	Ongoing pregnancy	
EPL (n/%)	607 (62.84%)	359 (37.16%)	966 (100.00%)
Ongoing pregnancy (n/%)	76 (1.21%)	6219 (98.79%)	6295 (100.00%)
Verification sample			
EPL (n/%)	621 (64.69%)	339 (35.31%)	960 (100.00%)
Ongoing pregnancy (n/%)	70 (1.22%)	5686 (98.78%)	5756 (100.00%)

*EPL*  early pregnancy loss

## Discussion

In this study, we collected the demographic and ultrasound findings for days 27–29 after ET and constructed a simple scoring system for the prediction of EPL, with AUCs of 0.884 and 0.878 in the training set and verification set, respectively. Point 5 had the highest predictive accuracy and was recommended as the cutoff value for clinical practice.

The process of achieving pregnancy through IVF is usually very difficult for an infertile woman. Thus, when the woman becomes pregnant by IVF, she is usually very anxious about the development of the embryo, even after the detection of cardiac activity but especially if an empty gestational sac or only a YS are detected. Generally, for patients who have well-developed embryos at the first routine TVS examination on days 27–29 after ET, the next ultrasound scan will usually be scheduled on day 45 after ET; however, for patients with embryos of uncertain viability, the recommendation is usually to have another TVS scan 7–10 days after the first scan to assess the development and viability of the embryo.

If the pregnancy outcome can be predicted in advance, the anxiety of the pregnant woman can be greatly relieved. Many models have been constructed to predict pregnancy outcomes effectively [13–15]. Among them, LR analysis has been the most commonly used [15, 16]. The overall goal of this study was to construct a simple and practical scoring system that is similar to the “Apgar score system” using simple demographic and ultrasound findings on days 27–29 after ET, as this would be easy for clinical application, especially for patients without sufficient assisted reproduction indicators when they return home after pregnancy. Through this system, we can inform patients with a specific probability of miscarriage after the first routine TVS examination, which may help reduce the patient’s anxiety and psychological burden and provide guidance for follow-up decisions.

**Fig. 2:**
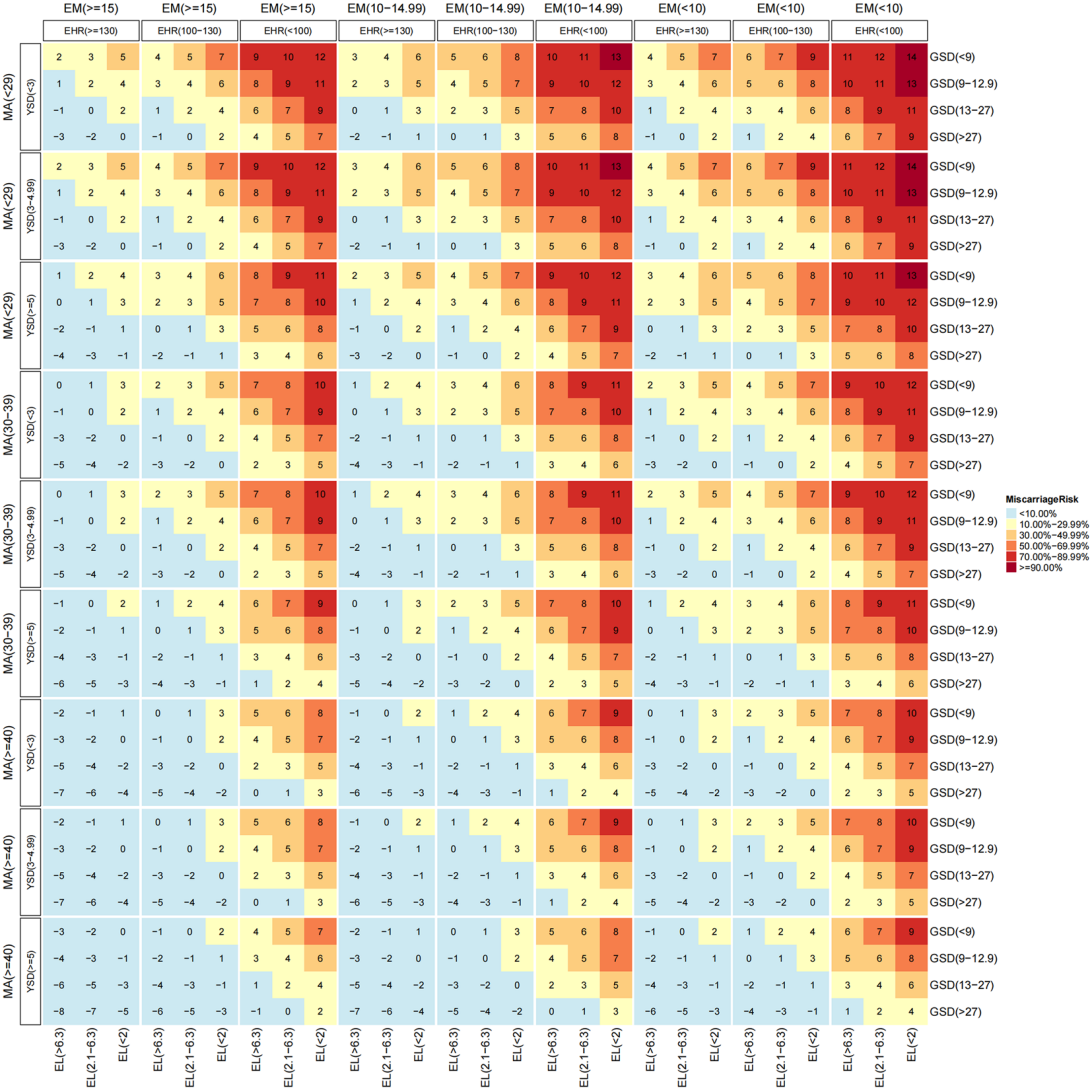
Risk of EPL in infertile women with an intrauterine singleton pregnancy based on the scoring system. *EPL* early pregnancy loss; *MA* maternal age (years); *EM* endometrium (mm); *GSD* gestational sac diameter (mm); *EL* embryonic length (mm); *EHR* embryonic heart rate (bpm); *YSD* yolk sac diameter (mm)

The findings of this study are consistent with the current knowledge that miscarriage is more likely with increasing MA [17], low hCG level [18], low EM thickness on transfer day [19] and the presence of IUH [20] and that miscarriage is less likely after the visualization of embryonic cardiac activity [15]. Accordingly, in our scoring system, a greater MA was associated with a higher score, while greater GSD, YSD, EL and EHR were all associated with lower scores. However, both small and large YSDs corresponded to higher scores. A higher score indicates a greater contribution to miscarriage. Thus, the possibility of miscarriage increased with the total score. A score of 5 offered the highest predictive accuracy in both the training set and verification set, and the corresponding miscarriage risk exceeded 30%, which was not a low risk for anxious IVF patients. Therefore, we recommend using a score of 5 as a clinical threshold for warning patients of this risk and providing more counselling about miscarriage. In practical applications, we hope that the false-positive rate (FPR) will be as low as possible, as this may lead to unnecessary medical treatment. A low FPR requires high specificity. In this system, the specificity of scores -8 to 0 was not satisfactory. Thus, this is a scoring system for determining the probability of miscarriage that could allow doctors and patients to know the risks in advance, but because miscarriage cannot be prevented, the outcome cannot be changed by this scoring system. The advantage of this scoring system is that it is easily transferable to clinical use, where both maternal and ultrasound variables are easily available and the calculation is simple. For example, in a woman with a MA of 38 years and who has an EM of 8 mm on transfer day, a GSD of 10 mm, a YSD of 2.2 mm, an EL of 2.5 mm and an EHR of 88 bpm by TVS on Day 28 after ET, the miscarriage score is 9, and the estimated risk of miscarriage is 70.67% (Fig. 2). Even though the results indicate a strong likelihood of EPL, there is still a significant risk that this is a false-positive finding. Thus, it is imperative to repeat the ultrasound scan for this patient 7–10 days later to confirm the viability of the embryo before performing any medical interventions. In contrast, if a patient does not present with any symptoms and has a low total score (for example, 0), no follow-up TVS scan is needed until Day 45 after ET.

A previous study [21] constructed a similar scoring system to predict pregnancy viability and achieved an AUC of more than 0.90. However, this study focused on natural conceptions, and the ultrasound variables and clinical characteristics collected in that study were collected within a time period of GA < 84 days. The measurement methods may also have differed from ours. However, in our study, all ultrasound parameters were collected at the same time on Days 27–29 after ET, and the specific EHR value was included, not just the presence or absence of embryonic cardiac activity. IUH was unexpectedly not included in the final system. We speculate that this may be because IUH occurs more frequently in pregnancies after IVF than in spontaneous conceptions [22], not only in women with EPLs but also with ongoing pregnancies.

We note that the sensitivity of this simple model was not ideal, which might indicate that for the IVF population, including only ultrasonic measurements and simple clinical indicators had limited predictive efficacy, and in further studies, relevant indicators for assisted pregnancy should be added to improve the predictive efficacy. A major limitation was that since there was no information on bleeding, abdominal pain or smoking history in our hospital's electronic medical record system during the study period, these indicators were not included in our system, but they might further improve the predictive performance if included. Since this system was derived from the IVF population, its application in the general population has yet to be validated.

In conclusion, we have developed a simple and practical scoring system that provides a probability for EPL based on the simple demographic and ultrasound findings obtained on days 27–29 after ET. This system is easy and simple for clinical use. Point 5 is recommended as the clinical threshold for warning patients of an EPL risk. When the predictive result is a high risk of EPL, repeated scans are recommended 7–10 days later to confirm the viability of the embryo. When the predictive result is a low risk of EPL and patients have no symptoms, the next examination can be performed on day 45 after ET.

## Supplementary Information

**Additional file 1: Table S1.** Performance of the scoring system in the verification sample.

## Data Availability

The data analysed during this study are included in the tables. The datasets used during the current study are available from the corresponding author on reasonable request.

## Abbreviations

EPL: Early pregnancy loss
IVF-ET: In vitro fertilization-embryo transfer
CI: Confidence interval
PPV: Positive predictive value
NPV: Negative predictive value
MA: Maternal age
YSD: Yolk sac diameter
GA: Gestational age
TVS: Transvaginal sonography
GSD: Gestational sac diameter
YS: Yolk sac
EL: Embryonic length
EHR: Embryonic heart rate
IUH: Intrauterine hematoma
HCG: Human chorionic gonadotropin
EM: Endometrium
LR: Logistic regression
AUC: Area under curve
FPR: False positive rate
