# Supplementary material for: A simple scoring system for the prediction of early pregnancy loss developed by following 13,977 infertile patients after in vitro fertilization
Source: Eur J Med Res. 2023 Jul 14;28:237. doi: 10.1186/s40001-023-01218-z (PMC10347825; doi:10.1186/s40001-023-01218-z)
Supplement: Supplementary file 1 — Additional file 1: Table S1. Performance of the scoring system in the verification sample. [file 40001_2023_1218_MOESM1_ESM.doc]

Table S1. Performance of the scoring system in the verification sample

| Sum of points | Sensitivity (%) | Specificity  (%) | PPV  (%) | NPV  (%) | ACC  (%) |
| --- | --- | --- | --- | --- | --- |
| -8 | 100.00% | 0.00% | 14.29% | -100.00% | 14.29% |
| -7 | 100.00% | 0.00% | 14.29% | 100.00% | 14.29% |
| -6 | 100.00% | 0.02% | 14.30% | 100.00% | 14.31% |
| -5 | 100.00% | 0.03% | 14.30% | 100.00% | 14.32% |
| -4 | 100.00% | 0.10% | 14.31% | 98.21% | 14.38% |
| -3 | 99.90% | 0.96% | 14.40% | 96.37% | 15.10% |
| -2 | 98.75% | 5.54% | 14.85% | 96.72% | 18.87% |
| -1 | 91.67% | 41.02% | 20.58% | 96.21% | 48.26% |
| 0 | 88.23% | 49.83% | 22.68% | 95.29% | 55.32% |
| 1 | 73.65% | 88.85% | 52.41% | 95.13% | 86.67% |
| 2 | 71.04% | 94.27% | 67.39% | 94.65% | 90.95% |
| 3 | 66.88% | 97.69% | 82.84% | 94.48% | 93.28% |
| 4 | 65.52% | 98.51% | 87.97% | 94.37% | 93.79% |
| 5 | 64.69% | 98.78% | 89.87% | 93.62% | 93.91% |
| 6 | 59.48% | 99.24% | 92.85% | 92.98% | 93.55% |
| 7 | 55.00% | 99.37% | 93.62% | 91.44% | 93.03% |
| 8 | 44.06% | 99.65% | 95.49% | 90.11% | 91.71% |
| 9 | 34.38% | 99.74% | 95.65% | 88.50% | 90.40% |
| 10 | 22.19% | 99.84% | 95.95% | 87.48% | 88.74% |
| 11 | 14.27% | 99.95% | 97.86% | 86.32% | 87.70% |
| 12 | 5.00% | 99.98% | 97.96% | 86.01% | 86.41% |
| 13 | 2.50% | 100.00% | 100.00% | 85.74% | 86.06% |
| 14 | 0.31% | 100.00% | 100.00% | 100.00% | 85.75% |
